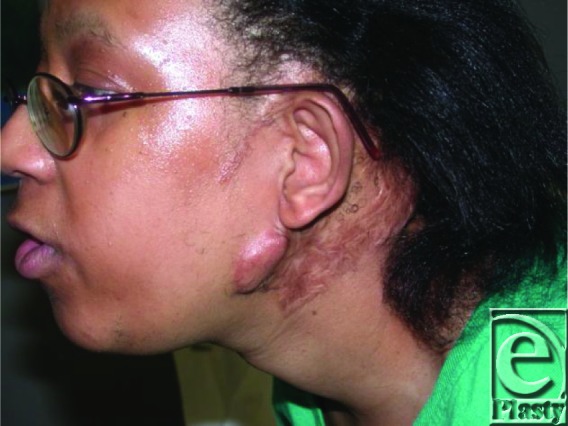# The Keloid Strikes Back

**Published:** 2012-05-24

**Authors:** Howard D. Wang, Raja Mohan, Nelson Goldberg

**Affiliations:** University of Maryland School of Medicine, Baltimore, MD

## DESCRIPTION

A 12-year-old African American woman presented to a plastic surgeon with a 1-year history of a slowly growing soft tissue mass behind her left ear. According to her, she had chicken pox and a residual pock mark behind that ear. As a result of irritation in that area from her glasses, she developed a cosmetically deforming keloid shown on the left. The keloid was subsequently excised, but 1 year later, she developed recurrence of the keloid that measured 11 × 4 cm. The recurrent keloid is pictured on the right.

## QUESTIONS

1. **What is the pathogenesis of a keloid?**
2. **What are the different treatment modalities for keloids?**
3. **How can the recurrence of keloids be prevented?**

## DISCUSSION

Keloids can be a difficult condition to manage primarily because of the high rate of recurrence associated with them. The patient described in this case developed multiple recurrences of her left postauricular keloid despite multiple excisions and radiation therapy. The picture appearing later was taken during the patient's last visit demonstrating the residual effects of multiple excisions and radiation therapy. She was 34 years old at the time, which is 22 years after the initial presentation. During this period of 22 years, the patient underwent 7 surgical excisions, 3 radiation treatments, and 3 corticosteroid injections.

Both keloids and hypertrophic scars are fibroproliferative disorders of the skin that results from aberrant wound healing. Clinically, they can be differentiated by the extent of scar involvement. Dense fibrous tissue that grows beyond the boundaries of the original wound and rarely regresses is a keloid. In contrast, hypertrophic scars lie within the boundaries of the wound and undergo regression after a static phase.^1^ Keloids are more commonly seen in individuals between the age of 10 and 30 and those of African, Asian, or Hispanic descent. The incidence of keloids can be as high as 16% in the African and Hispanic populations, which suggests the presence of a genetic component.^2^ Other risk factors that promote the development of keloids include mechanical force, wound infection, and foreign body reactions.^3^ Careful avoidance of these risk factors is important to prevent development of keloids after surgery or injury.

Despite extensive inquiries, the pathogenesis of keloids remains poorly understood. The current theories on keloid development often center on dysfunctional fibroblasts that lead to overproduction of type I procollagen and exhibit lower rates of apoptosis. In addition, the elevated levels of transforming growth factor β, platelet derived growth factor, and vascular endothelial growth factor found within keloids promote fibroblast proliferation and collagen production.^1^ In addition, a study by Granick et al^3^ showed that early telomerase activity may be responsible for the proliferation of keloids in their early stages.

A wide array of treatment modalities exists for management of keloids; however, no single optimal algorithm exists. Surgical excision is often used but should be combined with adjuvant therapy due to high rates of recurrence when used alone, up to 45% to 100%.^4^ Intralesional corticosteroids, primarily triamcinolone and radiotherapy can both be used as either monotherapy or adjuvant to surgical excision. The effectiveness of corticosteroid injection monotherapy has been reported to be 50% to 82%.^4^ The results of one prospective study suggest that triamcinolone alone and the combined treatment of surgical excision with adjuvant corticosteroids were similarly effective.^5^ The combination of surgery and radiotherapy achieves a success rate of 67% to 98%, but the risk of inducing malignancy for treating a benign lesion with radiotherapy is an important concern.^4^ Some authors have argued that the risk of carcinogenesis is very low when the correct dosage is used and adequate precautions are taken.^6^ Radiotherapy is favored in areas without underlying viscera such as the extremities. It should not be used in the pediatric population or pregnant women because of the theoretical risk of inducing malignancy.^7^ Other therapies for treating and preventing recurrence of keloids include topical 5-fluorouracil, imiquimod, silicone gel products, compression therapy, laser, and cryotherapy.

The decision of which therapy to be utilized often must be made on an individualized basis. In patients with a few or small keloid lesions, radical treatment with surgical excision plus adjuvant therapy such as radiation or corticosteroid may be used as the first-line treatment. The alternative method is a nonsurgical monotherapy such as corticosteroid, cryotherapy, laser, or antitumor agents. In patients with multiple or large keloids, a comprehensive discussion regarding the goals of treatment and expectations of the patient must be performed before initiating therapy. Treatment strategies include mass reduction surgery or symptomatic multimodal therapy consisting of a combination of the nonsurgical treatments including corticosteroid injections, cryotherapy, laser, antitumor agents, compression, and gel sheets. Recurrence of disease often requires switching to an alternative treatment modality.^4^ While the arsenal of therapies for the management keloids is quite large, it remains a complicated condition to treat with a high incidence of recurrence.

## Figures and Tables

**Figure:**
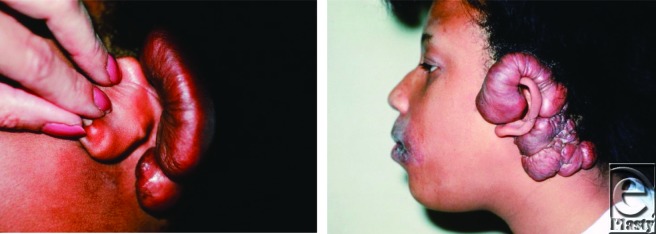


**Figure:**